# Prediction across healthcare settings: a case study in predicting emergency department disposition

**DOI:** 10.1038/s41746-021-00537-x

**Published:** 2021-12-15

**Authors:** Yuval Barak-Corren, Pradip Chaudhari, Jessica Perniciaro, Mark Waltzman, Andrew M. Fine, Ben Y. Reis

**Affiliations:** 1 Predictive Medicine Group, Computational Health Informatics Program, Boston Children’s Hospital, Boston, MA USA; 2 Division of Emergency and Transport Medicine, Children’s Hospital Los Angeles and Keck School of Medicine of the University of Southern California, Los Angeles, CA USA; 3 Harvard Medical School, Boston, MA USA; 4 Emergency Medicine, Boston Children’s Hospital, Boston, MA USA

**Keywords:** Epidemiology, Translational research

## Abstract

Several approaches exist today for developing predictive models across multiple clinical sites, yet there is a lack of comparative data on their performance, especially within the context of EHR-based prediction models. We set out to provide a framework for prediction across healthcare settings. As a case study, we examined an ED disposition prediction model across three geographically and demographically diverse sites. We conducted a 1-year retrospective study, including all visits in which the outcome was either discharge-to-home or hospitalization. Four modeling approaches were compared: a ready-made model trained at one site and validated at other sites, a centralized uniform model incorporating data from all sites, multiple site-specific models, and a hybrid approach of a ready-made model re-calibrated using site-specific data. Predictions were performed using XGBoost. The study included 288,962 visits with an overall admission rate of 16.8% (7.9–26.9%). Some risk factors for admission were prominent across all sites (e.g., high-acuity triage emergency severity index score, high prior admissions rate), while others were prominent at only some sites (multiple lab tests ordered at the pediatric sites, early use of ECG at the adult site). The XGBoost model achieved its best performance using the uniform and site-specific approaches (AUC = 0.9–0.93), followed by the calibrated-model approach (AUC = 0.87–0.92), and the ready-made approach (AUC = 0.62–0.85). Our results show that site-specific customization is a key driver of predictive model performance.

## Introduction

Driven by advances in machine learning and large quantities of data accumulating in healthcare settings worldwide, recent years have witnessed a dramatic proliferation in the development and application of predictive models in medicine^1,2^. These models predict a range of outcomes, including in-hospital mortality, cancer, suicide, abuse, and new-onset dementia^3–9^. A major challenge in the development of predictive models is cross-site generalizability. Even when standardized protocols are in place to enable data and model interoperability (e.g., HL-7, DICOM, or SMART and FHIR)^10,11^, there is still no guarantee that an algorithm developed at one site will work well at another.

Medical settings can vary in numerous ways, including case-mix^12^, demographic distributions^13^, care processes^14^, coding practices^15^, and use of laboratory tests^16^, among others^17^. Diagnostic tests that are routinely used at one site may be scarce at another^18^, hence the significance of such tests in a prediction model is expected to vary between sites. Furthermore, some information may only be available at some sites: for example, the Children’s Hospital Early Warning Score^19^ is only available at some pediatric hospitals. Thus, a model that relies on this risk score will not be transportable to sites that do not collect it, and a model built without access to this score may miss important information if applied at a pediatric site in which this information is collected. These differences can undermine model generalizability and transportability across sites, serving as a major barrier to adoption^20^.

Four main approaches are commonly used today to address the challenges of prediction across multiple sites: (1) Training and testing a model at each site separately in order to build *site-specific* models^11,21–24^; (2) creating a centralized database of data from all sites in order to build a single *uniform* model^25,26^; (3) applying a *Federated Learning* (FL) approach in which the model is trained collaboratively at each site, sharing model parameters but not medical data across sites^27,28^; and (4) when customization is not feasible or when little variation exists between sites, a *ready-made* model that was trained at one or a few sites, can be used across all sites. This last approach is common practice with clinical risk scores such as the Centor risk score for Group-A Streptococcus pharyngitis or the PECARN algorithm for managing children with traumatic brain injury, to name a few^29–31^. It is also commonly used in commercial deep-learning models such as the FDA-approved AiDoc tool for the identification of intracranial bleeding in computed tomography (CT) scans^32^. There are also hybrid approaches that combine elements derived from these different approaches^33^.

We sought to compare these different approaches and to examine their strengths and weaknesses, through a case study of a hospitalization prediction model, implemented across three diverse real-world healthcare settings: Boston Children’s Hospital (BCH), Children’s Hospital Los Angeles (CHLA), and South Shore Hospital (SSH) Long-term boarding in emergency departments (ED) contributes to overcrowding, a pervasive problem in the United States hospitals^34,35^. In the current model of care, patients in the ED are managed in a serial fashion, beginning with registration, followed by triage, diagnosis, and treatment. Only toward the end of the ED encounter, based on the information collected during the encounter, a decision is made about whether the patient should be admitted or discharged. The serial approach to care leads to inefficiencies in assigning the newly admitted patient to a specific bed or room in an inpatient department, which in turn can contribute to prolonged ED boarding times and overcrowding. Early prediction of ED patient disposition can assist in shortening boarding times by facilitating earlier requests of inpatient beds, or in shortening the overall ED length-of-stay by informing ED staff about patients likely to be discharged so they can prioritize treatment^36^.

To address this issue, we previously developed the prediction of patient placement system^36–38^, which utilizes routinely collected clinical and administrative information to accurately predict patient disposition (AUC = 0.92). We found that these models were influenced by local care practices. For example, at one site, order for (or lack of) a blood test for calcium was very predictive of hospitalization—not because calcium levels by themselves were predictive of hospitalization, but rather because calcium was taken as part of a routine set of tests for the more critically ill patients at that specific institution. At another institution, patients for whom height was measured were more likely to be admitted. Again, this was not the result of an association between stature and likelihood of admission, but rather since at that particular care setting, height was measured almost exclusively for patients requiring chemotherapy or patients with a severe eating disorder. These local practices can challenge the generalizability of such models and the ability to implement the model in other settings.

In this study, we provide a general framework for developing prediction models across multiple settings. We implement our previously developed hospitalization-prediction model at three diverse clinical sites and use this case study as an opportunity to compare different cross-site implementation strategies based on the accepted methods mentioned above.

## Results

### Setting

During the study period, 197,683 unique patients visited the three included EDs in 300,504 separate encounters (Supplementary Fig. 1). Of these, 11,542 visits (3.8%) were excluded due to an outcome other than hospitalization/discharge—for example: left against medical advice, left without being seen, death, or transfer to another facility. Overall, 288,962 visits by 191,449 different patients were included in the analysis. Of the 288,962 included ED visits, 48,651 (16.8%) were admitted to the hospital and 240,311 (83.2%) were discharged home. The median volume of ED visits per day was 167 (range: 72–267) at BCH, 239 (67–363) at CHLA, and 245 (118–312) at SSH. The overall hospitalization rate was 19.1% at BCH, 7.9% at CHLA, and 26.9% at SSH (Table 1). The rate did not vary significantly over the course of the study, but weekday variations were noted with a lower admission rate during the weekend compared to the middle of the week (*p* < 0.05, Supplementary Fig. 2).

**Table 1:** Comparison of study sites by basic demographics and ED statistics.

	BCH	CHLA	SSH
Total visits	76,218	124,048	88,696
Total patients	52,075	76,949	62,425
Admission rate	19.1% (*n* = 14,979)	7.9% (*n* = 9843)	26.9% (*n* = 23,829)
Gender (females)	47% (*n* = 37,319)	46% (*n* = 57,459)	53% (*n* = 47,049)
Age in years (median and IQR)	6.4 (2.1–13.4)	4.5 (1.6–9.4)	46.3 (22.9–67.4)
ED length of stay in hours (median and IQR)	3.5 (2.0–13.3)	2.4 (1.4–3.8)	5.0 (3.0–9.0)

### Risk factors for admission

As expected, the average age at BCH and CHLA (both pediatric centers) was much lower than that of the SSH general-population ED (6.4 and 4.5 vs. 46.3 years). Whenever older patients did visit the CHLA ED, they were more likely to be hospitalized than the younger patients (>20% admission rate for patients over the age of 19 vs. <10% for patients under the age of 15). The reasons for visits also differed across the sites, with chief complaints such as fever, abdominal pain and/or vomiting, and cough and/or breathing difficulties ranking as the most common complaints at BCH and CHLA, while complaints such as fall, chest pain, shortness of breath, and abdominal pain ranking as the most common complaints at SSH. The median length of stay in the ED ranged from 2.7 h in the pediatric sites (IQR 1.7–4.3) to 5.0 h (IQR 3.0–9.0) at the general ED (SSH). On average, across all sites, admitted patients stayed longer in the ED than discharged patients. From 3.2–4.2 h longer at BCH and CHLA to 13.9 h longer at SSH.

At all sites, the ESI acuity score was predictive of hospitalizations, with a 0–2% admission rate across sites for the least acute triage levels (ESI 4–5) and 79–93% for patients with the most acute triage score (ESI 1). In two of the sites (BCH and SSH), a history of prior admissions was highly predictive of future admission, with a previous admission rate of >50% of prior ED visits associated with a 90% likelihood of admission in the index visit. In the pediatric sites, the number of laboratory tests ordered in the first 60 min was also predictive of admission, while in the general (adult) ED, patients who had an electrocardiogram (ECG) within the first hour were 2.6 times more likely to be admitted than those without an ECG (50% vs. 19% admission rate). Further description of the risk factors can be found in Supplementary Tables 1–3 which show the top features at each site, and in Supplementary Table 4 which shows the features selected by the multivariate model.

**Fig. 1:**
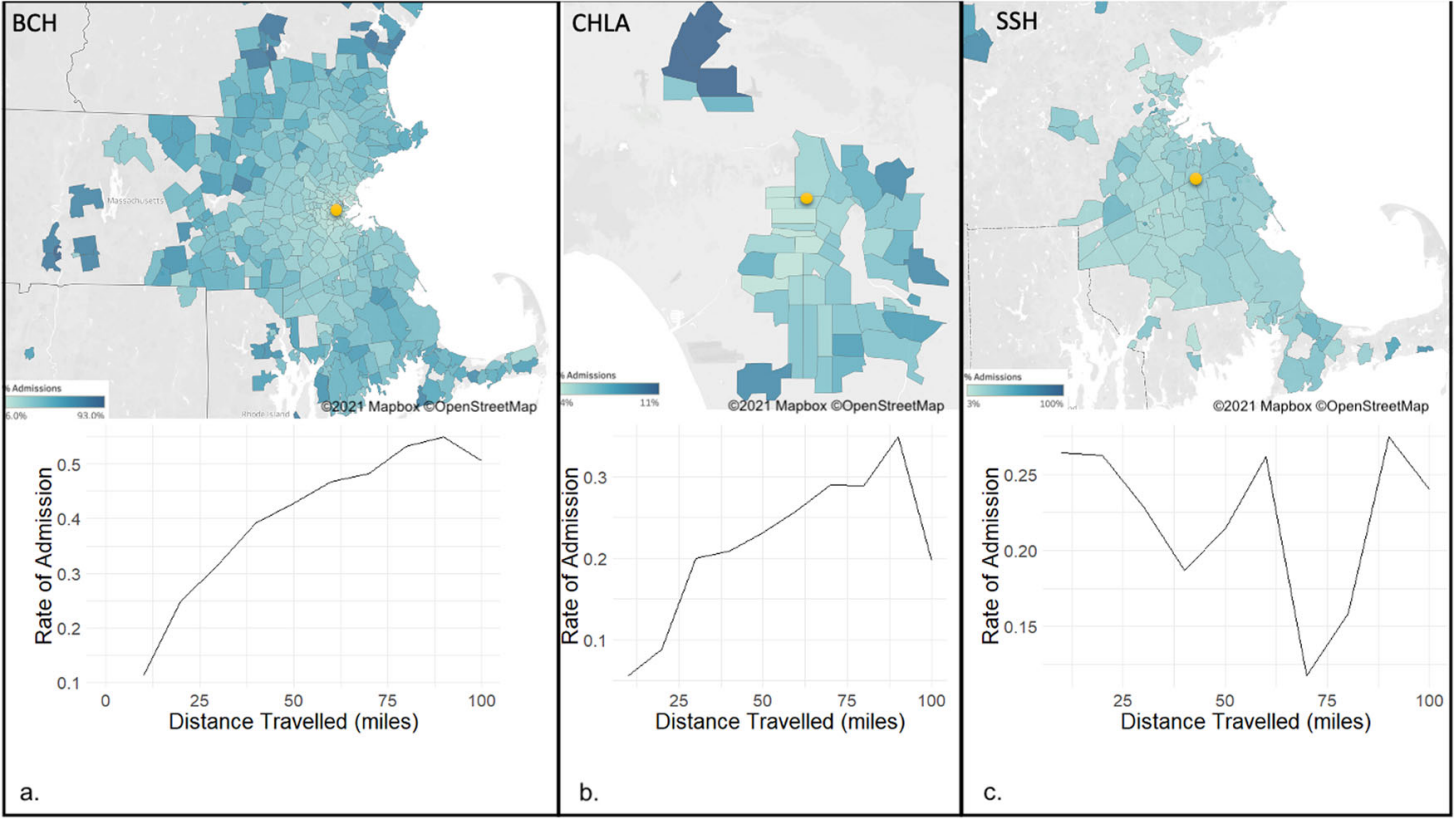
Admission rate by zip-code and miles traveled. In both BCH and CHLA patients that come from farther away were more likely to be admitted. In contrast, no such correlation was found for SSH. The maps were generated using Tableau software (https://www.tableau.com) and using ^©^OpenStreetMap data. **a** Results for BCH; **b** results for CHLA; **c** results for SSH.

The distance patients traveled to reach the ED was also predictive of admission (Fig. 1). At BCH, a national referral center, this effect was most prominent and the rate of admission increased from 11% for those traveling less than 10 miles to about 50% for those traveling 100 miles. At CHLA the rate increased from 6% to about 30% as the traveling distance increased. At SSH, while some zip-codes were associated with an admission rate of over 70% and other zip-codes were associated with an admission rate as low as 1.6%, these were not particularly related to the distance from the hospital.

### Multivariate model

**Fig. 2:**
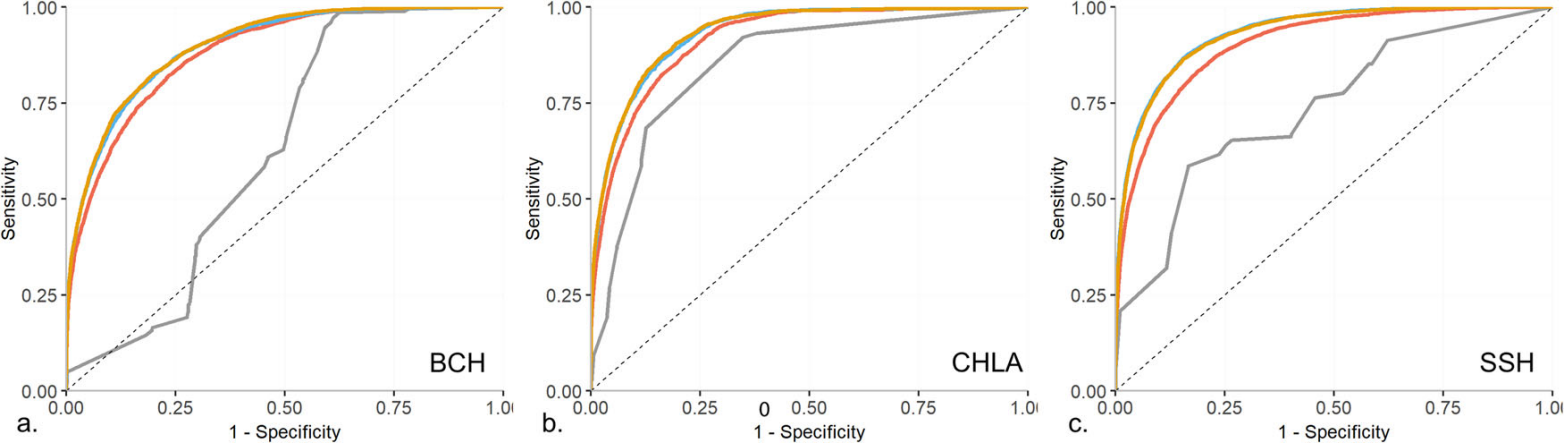
ROC plots comparing the performance of the different modeling approaches in each study site. Each chart shows four ROC plots: red for the calibrated model, gray for the ready-made model, blue for the site-specific model, and orange for the uniform model. **a** Results for BCH, the hybrid model developed using CHLA data. **b** Results for CHLA, hybrid model developed using BCH data. **c** Results for SSH, hybrid model developed using BCH data.

The top 20 features selected at each of the sites are shown in Supplementary Table 4. Model performance varied by the approach used, with the best results achieved using the *site-specific* and *uniform* approaches, followed by the *calibrated-model* approach and the *ready-made* approach (Fig. 2, Table 2). Overall, the *site-specific, uniform*, and *calibrated* model approaches all achieved relatively good performance with AUCs around 0.90 (0.87–0.93). In contrast, in most cases, the *ready-made* approach achieved an AUCs of around 0.6 (0.62–0.65), with the best performance achieved when the model was built using BCH data and applied at CHLA (AUC = 0.85). A summary of these findings can be found in Supplementary Fig. 3.

**Table 2:** Predictive performance for all model types and all sites.

Site	Model type	Specificity (%)	Sensitivity (%)	PPV (%)	NPV (%)	AUC
BCH	Site-specific	90	68	61	93	0.9
	Uniform	90	68	62	92	0.9
	Ready-made (CHLA)	90	10	19	81	0.62
	Ready-made (SSH)	90	33	43	85	0.64
	Calibrated (CHLA)	90	61	59	91	0.87
	Calibrated (SSH)	90	64	60	91	0.88
CHLA	Site-specific	90	76	39	98	0.93
	Uniform	90	77	40	98	0.93
	Ready-made (BCH)	90	53	31	96	0.85
	Ready-made (SSH)	90	29	20	94	0.62
	Calibrated (BCH)	90	72	38	97	0.92
	Calibrated (SSH)	90	72	38	97	0.92
SSH	Site-specific	90	80	74	93	0.93
	Uniform	90	78	74	92	0.93
	Ready-made (BCH)	90	30	52	79	0.74
	Ready-made (CHLA)	90	32	53	79	0.65
	Calibrated (BCH)	90	72	72	90	0.91
	Calibrated (CHLA)	90	73	72	90	0.91

The site-specific model was trained and validated on data from each site. The hybrid model used features selected at BCH and then trained with data from each site. The uniform mode’ was built and trained using BCH’s data and then applied on the other sites’ data “as is” and without adjustment of the model’s coefficients.

## Discussion

The results of this study show that site-specific customization is a key driver of predictive model performance. The best performance was achieved when the models were trained using site-specific data (AUC of 0.87–0.94) versus when applying a *ready-made* model developed using only one site (AUC of 0.62–0.85). The drop in performance was less pronounced when transferring the model from one pediatric ED (BCH) to another pediatric ED (CHLA).

The study further showed that certain factors, such as the history of prior admissions, patient age, triage score, the room in which a patient was placed in the ED, or the number of lab tests ordered for the patient, are common risk factors for admission across all sites, while other features, such as the involvement of particular team members, zip codes associated with higher rates of admission, or specific types of laboratory tests ordered are hospital-specific. Some factors are common to some of the sites but not to others (e.g., the order of venous blood gases was predictive of admission at the two pediatric sites and not at SSH).

Studies that evaluate the performance of predictive models at multiple sites often involve objective tasks that are easy to replicate, such as image processing or analysis of laboratory tests^25,26,28,39^. In this respect, the current study addresses a non-trivial prediction task: the outcome is subjective and provider-dependent, the variables include non-objective findings such as orders given, and the overall admission rate is influenced by the local patient population. In the present study, the admission rate ranged from 8% at CHLA to 27% at SSH. Thus, it is not surprising that models that included site-specific training performed better than the more generalized models. Studies that involve a more transferable prediction task, such as image processing of radiology or pathology images, would most likely work well without site-specific customization.

The approaches described in this study have various benefits and drawbacks. The *site-specific* approach is relatively easy to implement and is optimized to local site characteristics^40^. However, it does not utilize information from all sites and often requires skilled personnel and data availability at the target site for feature extraction and model training. The *uniform* model approach utilizes all available data, but poses a significant privacy risk, as data leave the institution into a centralized data repository. Furthermore, this approach may miss some of the site-specific nuances during the data aggregation process^41^. The *ready-made* approach has the advantage of the simplicity of implementation. Also, it is often easier for end-users such as clinicians to comprehend. Nonetheless, this approach does not benefit from the large sample size that can be obtained from multiple sites and it has no site-specific customization, hence its predictive performance is expected to be poorer than the other approaches for most prediction tasks. This approach is often used only as a secondary step after a model has initially been developed in a multi-site setting using one of the other approaches. The *calibrated* model approach, by combining the *ready-made* and *site-specific* approaches, enables both simplicity and customizability, but only to a limited extent and without taking into account site-specific features. Nonetheless, we found that the performance of this calibrated model was comparable to the performance of the other top-performing models (i.e., site-specific and uniform models). The performance would likely further improve if the feature-selection process would be done using multiple sites and not just one (i.e., training on three sites and calibrating on the fourth). While not implemented in the present study, the *federated learning* approach also holds great potential. It provides the benefits of the *site-specific* and *uniform* approaches in terms of customization, sample size, and generalizability, but without the inherent privacy risks of these approaches. However, it is a complex method that requires skilled staff at all sites, more computational resources, and it is more difficult to infer clinical insights from these models as the clinical data are not shared. Beyond these inherent limitations of federated learning, in the current study, we chose not to apply this method as its added value is limited in cases where there are relatively few variables, few sites, and many observations at each site.

Based on these considerations, we provide a framework for future development and implementation of prediction models across multiple sites. This framework is especially relevant for predictions that involve subjective outcomes and outcomes dependent on custom care practices. We recommend the following steps: Starting with the site-specific approach, one can measure the top benchmark for model performance as well learn the similarities and differences in risk factors across sites. Using the *ready-made* approach, the minimally viable solution can be obtained and the lowest bar for model performance can be delineated. The *calibrated* and *uniform* approaches are often more usable and easier to implement as they require fewer features to extract (in the former) and less site-specific development (in the latter), thus it is useful to estimate this tradeoff in overall performance for these approaches as well. Comparing the performance of all approaches and for all sites can help estimate how the model will perform in future sites as well. In order to apply this framework, all sites must collect a similar set of variables in machine-readable form. To determine the ‘must-have’ variables, the model should first be trained at one of the sites, and the most important types of variables may be identified. We believe that the results of our analysis, together with the strengths and weaknesses that we highlight for each method, will be of interest to researchers embarking upon a multi-site prediction project, even when the implementation of the full framework is not possible.

This study has several limitations. First, all models required pre-processing of the data which may be challenging for some healthcare providers. Features such as the count of the number of heart-rate measurements, the distance between the patient’s home address and the hospital, or the ratio of prior admissions to visits may not be readily available in other sites and thus require some manual processing. However, as this challenge is not unique to the current scenario, perhaps SMART applications will one day allow automatic pre-processing of the data. Second, the matching of equivalent variables between sites was also time-consuming for this project and may not be applicable in other predictive tasks or with more sites involved. Nonetheless, this task is already being addressed by platforms such as FHIR and I2B2^10,42^. In addition, by applying feature selection we can limit the extent of this matching process. Of note, while our model supports FHIR and was originally implemented at BCH with FHIR since FHIR was not supported at the other two sites, it was not used in the current study. Lastly, this study does not provide an exhaustive review of all possible multi-site implementation strategies. Furthermore, we only studied one type of modeling technique (XGBoost), and our results may not be applicable to types of models. We highlight the main elements of such strategies and the importance of site-specific customization, but further studies will be needed to explore other approaches as well.

In conclusion, in this study, we provide a framework for evaluating the use of predictive algorithms across multiple sites. We demonstrate the strengths and weaknesses of different approaches for implementation and the insights that can be gained on a model’s generalizability. The proposed framework could serve as a guide for multi-site prediction scenarios, supporting the widespread implementation of predictive models in medicine.

## Methods

### Study design

We conducted a retrospective cross-sectional analysis of all ED visits at three medical centers: Boston Children’s Hospital (BCH), Children’s Hospital Los Angeles (CHLA), and South Shore Hospital in Weymouth, MA (SSH). Boston Children’s Hospital is a 415-bed tertiary care children’s hospital with an annual ED volume of approximately 60,000 visits. Children’s Hospital Los Angeles is a 495-bed tertiary care children’s hospital with an annual ED volume of approximately 94,000 visits. South Shore Hospital is a 396-bed community general hospital with an annual ED volume of approximately 100,000 adult and pediatric visits. We chose the three hospitals to provide some elements of diversity in terms of geography, age, and community vs. academic centers.

One year of data was analyzed from each site: Data from BCH and CHLA were available from January 2017 through December 2017, while data from SSH were available from July 2017 through July 2018. Analyses included all visits with an outcome of “discharge to home” or “admission to inpatient service.” All visits resulting in other dispositions were excluded. The study was approved by the Institutional Review Boards of all three participating hospitals.

### Data collection

For each visit, we collected information on patient demographics, triage emergency severity index (ESI) score^43^, mode of arrival, distance traveled vital signs, anthropometrics (height, weight, body mass index), medications ordered, laboratory tests ordered, radiology tests ordered, pain scores, risk scores such as the pediatric early warning score^44,45^, problem lists in the EHR previously documented by clinicians, and history of prior visits and admissions. Only structured variables were used. We obtained data from the EHRs in use at each institution. CHLA and BCH use Cerner EHR, and SSH uses EPIC EHR. In sites where information on the distance patients traveled was not available, we calculated this distance by using a database of distances between ZIP codes downloaded from the National Bureau of Economic Research website^46^. This study only included data that are routinely collected and that were available within the first 60 min of the ED encounter. Missing variables were used as features (e.g., lack of available blood tests in an encounter was an indication that no blood tests were taken within the first hour of the encounter).

### Model development

**Fig. 3:**
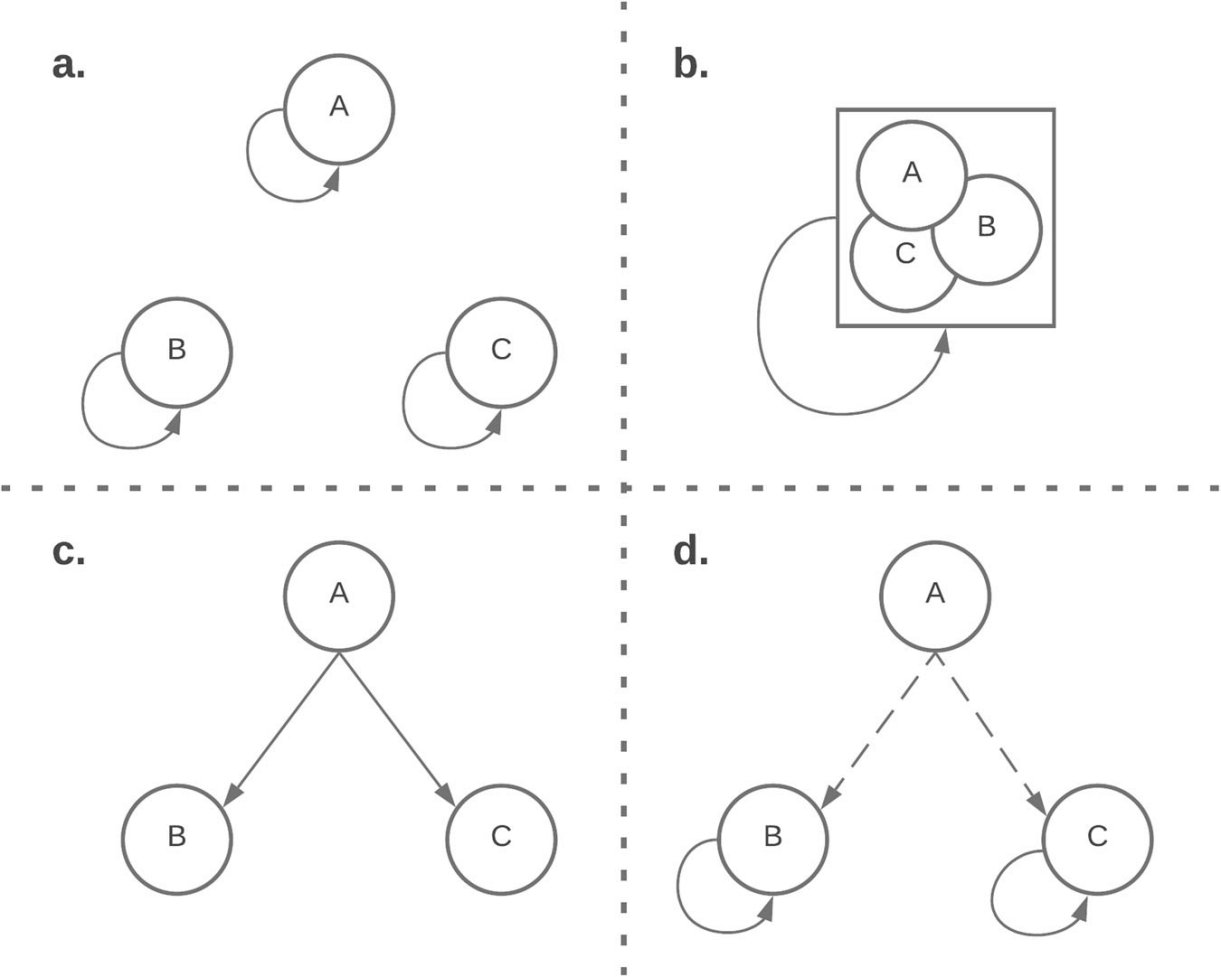
Summary of the four multi-site prediction strategies. **a** Site-specific model: three different models were generated using the same R code where each model was trained and validated using site-specific data. **b** Uniform model: one model was generated using the data from all sites combined. **c** Ready-made model: a model was trained at one site and then applied and validated at the other sites. **d** Calibrated model: one site was used for feature selection and the two remaining sites were used to create a customized model based on these features.

Four approaches were used for the development of the admission–prediction model at the different sites (Fig. 3): (1) In the *site-specific* approach, separate prediction models were developed and validated at each of the three participating sites. Each site’s encounters were randomly divided into two subsets, 70% used for model development (training) and 30% used for model validation (testing). Specific features and specific coefficients were identified for each of the sites. (2) In the *uniform model* approach, data from all sites were incorporated into a single large centralized dataset. This dataset was then randomly divided into 70% training and 30% testing sets that were used for model derivation and validation as in the site-specific approach, but only a single model was derived for all sites. (3) In the *ready-made* approach, the model was developed at one site and then applied and validated “*as-is*” at the other two sites (each time choosing a different site for model development). (4) In the *calibrated model* approach, we combined elements of the above approaches into a single approach similar to federated learning: the model was developed at one “training” site in order to select the top-20 features. These features were then passed to the two other validation sites where new coefficients were calculated using the site-specific training data. The resulting model was validated on each site’s testing set. As in the federated learning approach, no clinical data were shared between the sites—only the list of selected features. However, unlike federated learning, the weights of the model were not shared between sites.

All models were developed using the XGBoost package of the R statistical platform^47,48^. XGBoost was chosen based on our prior experience with similar prediction tasks, where XGBoost was found to be superior and more efficient than Random Forest, Naive Bayes, SVM, and Decision Trees. The full model configuration, and the R code used to build the model, can be found in the Supplementary material (Supplementary Note 1). Feature selection was conducted to pare down thousands of available features. This was accomplished by 5-fold repeat random sampling of a small portion of the training set containing 1000 observations, building an XGBoost model using this small sample, and then identifying the top 20 features selected by these sub-models. An XGBoost model was then built using the entire training set incorporating the 20-features identified in the feature-selection process. This model was validated on the validation set and a receiver operating characteristic curve was derived.

### Reporting summary

Further information on research design is available in the Nature Research Reporting Summary linked to this article.

## Supplementary information

Supplementary Information

Reporting Summary

## Data Availability

The data that support the findings of the study are available from the corresponding author upon reasonable request.

## Code availability

The source code is provided in the Supplementary material.
