# Supplementary material for: Prediction across healthcare settings: a case study in predicting emergency department disposition
Source: NPJ Digit Med. 2021 Dec 15;4:169. doi: 10.1038/s41746-021-00537-x (PMC8674364; doi:10.1038/s41746-021-00537-x)
Supplement: Supplementary file 1 — Supplementary Information [file 41746_2021_537_MOESM1_ESM.pdf]

Supplementary Materials

Supplementary Note 1

```
getTopFeaturesUsingXGBoost <- function(training.data, num_rounds = 5, sample_size = 1000,
params)
{
  selected_features = c()
  for (i in 1:num_rounds) {
    # sub sample the training set
    idxs = sample(1:nrow(training.data),sample_size,replace=F)
    training <- convertToXGBoostMatrix(training.data[idxs,])
    # build the model and find which were the top features used
    xgb.fit.small <- xgb.train (params = params, data = training, nrounds = 10, watchlist =
list(train=training), print_every_n = 10, early0_stopping_rounds = 10, maximize = F , eval_metric =
"error", verbose=0)
    selected_features = c(selected_features, getBestParams(xgb.fit.small, count = 20))
  }
  selected_features = unique(selected_features)
  return(selected_features)
}


buildAndValidateXGBoost <- function(training, testing, params)
{
  # find the nrounds parameter (equivalent to number of trees to grows in Random Forest)
  xgbcv <- xgb.cv( params = params, data = training, nrounds = 100, nfold = 5, showsd = T, stratified = T,
                   print_every_n = 10, early_stopping_rounds = 40, maximize = F)
  best_nrounds = xgbcv$best_iteration;
  xgb.fit <- xgb.train (params = params, data = training, nrounds = best_nrounds, watchlist =
                        list(train=training, val=testing), print_every_n = 10, early_stopping_rounds = 10,
                        maximize = F , eval_metric = "error")

  # validate performance on the testing set
  xgb.pred <- predict (xgb.fit, testing)
  true_outcome = getinfo(testing, 'label')
  xgb.roc.res = roc(true_outcome, xgb.pred)

  return(list(training, testing, xgb.fit, xgb.pred))
}


TRAIN_SIZE = 0.7

params = list(booster = "gbtree", objective = "binary:logistic", eta=0.3, gamma=0, max_depth=6,
min_child_weight=1, subsample=1, colsample_bytree=1)

# Select top-20 features
selected_features.ssh = getTopFeaturesUsingXGBoost(site_data.ssh[['training']])
selected_features.bch = getTopFeaturesUsingXGBoost(site_data.bch[['training']])
selected_features.chla = getTopFeaturesUsingXGBoost(site_data.chla[['training']])


# re-build the model using the best selected features
```

```
xgboost.ssh = buildAndValidateXGBoost(site_data.ssh[['training']][, c(selected_features.ssh, 'isCase')],
                                      site_data.ssh[['testing']][, c(selected_features.ssh, 'isCase')], params)
xgboost.bch = buildAndValidateXGBoost(site_data.bch[['training']][, c(selected_features.bch, 'isCase')],
                                      site_data.bch[['testing']][, c(selected_features.bch, 'isCase')], params)
xgboost.chla = buildAndValidateXGBoost(site_data.chla[['training']][, c(selected_features.chla, 'isCase')],
                                      site_data.chla[['testing']][, c(selected_features.chla, 'isCase')], params)
```

Supplementary Table 1 - Top risk factors associated with hospitalization for the BCH site.

Feature	Cases	Controls	OR	95% CI
Piperacillin-tazobactam	109	1	466.1	[65.07 - 3339.31]
Admission ratio > 50%	2,272	191	50.9	[43.84 - 59.03]
Drugs count >= 6	50	5	42.8	[17.05 - 107.25]
Arrival mode to ED by Air transport	74	9	35.2	[17.6 - 70.25]
Electrolytes (Na, K, Cl), Whole Blood	62	8	33.1	[15.87 - 69.22]
Resp rate documented >= 5 times	435	60	31.0	[23.66 - 40.64]
Heart rate documented >= 5 times	469	68	29.5	[22.86 - 38.06]
Lactic Acid, Whole Blood	527	78	28.9	[22.76 - 36.67]
Medication by intravenous drip	120	18	28.5	[17.37 - 46.8]
Dextrose 5% with 0.9% NaCl 1,000 mL	66	10	28.2	[14.51 - 54.9]
Blood Gas, Venous	1,429	244	25.0	[21.84 - 28.72]
Differential, Manual	57	10	24.4	[12.45 - 47.74]
Dextrose 5% with 0.9% NaCl 500 mL	157	28	24.0	[16.03 - 35.86]
Children's hosp early warning score >=5	448	89	21.5	[17.13 - 27.04]
WB Profile - IonCa, Na, K, Cl, Glu	360	72	21.4	[16.59 - 27.56]
Calcium, Ionized	77	16	20.6	[12.01 - 35.27]
CT-Head	61	13	20.1	[11.02 - 36.53]
Pancreatitis	51	11	19.8	[10.33 - 38.05]
Bench ABORh[¥]	64	15	18.2	[10.4 - 32.02]

¥ Bench ABORh - cross matching for blood transfusions.

Supplementary Table 2 - Top risk factors associated with hospitalization for the CHLA site.

Feature	Cases	Controls	Odds Ratio	95% CI
ED.ROOM	51	1	593.0	[81.9 - 4292.2]
Pain score (count=10)	335	20	194.8	[124 - 306]
Triage score = 1	181	13	161.9	[92.2 - 284.4]
(Stat)XR Chest AP PORTABL	49	7	81.4	[36.9 - 179.8]
Pain score (count=8)	90	16	65.4	[38.4 - 111.4]
(Stat)Chest AP PORTABLE X	56	10	65.1	[33.2 - 127.7]
Pain score (count=9)	64	13	57.2	[31.5 - 104]
Pain score (count=7)	98	22	51.8	[32.6 - 82.3]
(Stat)CSF Glucose	60	22	31.7	[19.4 - 51.7]
(Stat)CSF Protein	60	22	31.7	[19.4 - 51.7]
(Stat)CSF Culture	62	23	31.3	[19.4 - 50.6]
(Stat)CSF Cell Count	61	23	30.8	[19.1 - 49.9]
(Stat)Culture Blood Aerobic #1	45	21	24.9	[14.8 - 41.9]
Pain score (count=6)	146	69	24.6	[18.5 - 32.8]
Labs (count=10)	96	48	23.3	[16.4 - 32.9]
(Stat)Anaerobic Blood Culture	41	23	20.7	[12.4 - 34.6]

(Stat)Blood Culture	200	114	20.4	[16.2 - 25.7]
(Stat)Blood Bank Collection Ty	206	119	20.1	[16 - 25.3]
(Stat)Type & Screen	201	117	20.0	[15.9 - 25.1]

Supplementary Table 3 - Top risk factors associated with hospitalization for the SSH site.

Feature	Cases	Controls	OR	95% CI
CEFEPIME IVPB	96	-	NA	NA
heparin (porcine)	52	-	NA	NA
PANTOPRAZOLE INFUSION	60	1	168.9	[23.4 - 1218.6]
CPAP/BIPAP (NIPPV) -	99	2	139.3	[34.4 - 564.9]
Cardiac Catheterization	56	2	78.8	[19.2 - 323]
etomidate (AMIDATE)	52	2	73.2	[17.8 - 300.5]
Arterial Blood Gas with Electrolytes	169	9	52.8	[27 - 103.4]
propofol (DIPRIVAN) infusion - ADS Override Pull	71	4	50.0	[18.2 - 136.8]
heparin	49	3	46.0	[14.3 - 147.5]
HEPARIN SODIUM (PORCINE)	59	4	41.5	[15.1 - 114.3]
count_TransfusionAdministration = 1	49	5	27.6	[11 - 69.2]
BB Previous History Check	97	10	27.3	[14.2 - 52.4]
Respiratory.distress	83	9	26.0	[13 - 51.6]
count_BloodBank = 1	70	8	24.6	[11.8 - 51.2]
VANCOMYCIN	96	11	24.6	[13.2 - 45.8]
Blood Culture, Peripheral #2	378	45	23.6	[17.3 - 32.2]
Blood Culture, Peripheral #1	380	47	22.8	[16.8 - 30.8]
midazolam (VERSED)	104	13	22.5	[12.6 - 40.1]
Cellavision Differential	67	9	21.0	[10.4 - 42]
admission_ratio > 50%	3332	452	20.7	[18.8 - 22.9]

Supplementary Table 4 - Top 20 features identified by each site's model, ordered with features common to all sites listed first.

BCH	CHLA	SSH
Admission ratio	Admission ratio	Admission ratio
Age group	Age group	Age group
Triage score	Triage score	Triage score
ED room type	ED room type	ED room type
Number of labs ordered	Number of labs ordered	Number of labs ordered
Race	Race	
Prior admissions (count)	Prior admissions (count)	Prior admissions (count)
Venous blood gasses	Venous blood gasses	
CBC with differential	CBC with differential	
Heart rate (times taken)		Heart rate (times taken)
Heart rate (maximal value)		Heart rate (maximal value)
IV placed		IV placed
ED chief complaint		ED chief complaint
**BCH unique**	**CHLA unique**	**SSH unique**
Arrival mode to the ED	Pain score (times taken)	Teams involved (count)
Medical problems (count of)	Total prior visits	Comprehensive metabolic panel taken
Respiratory rate (max value)	Urinalysis	Medical problem = 'other'
Patient address (state)	Oxygen saturation (SD)	"Registered nurse"
Month in year	Oxygen saturation (min)	Total number of prior visits
Heart rate (SD)	Time until first medication	Lactic acid, taken
Miles traveled	Pain score (max)	"Physician Assistant"
	Oxygen saturation (mean)	Chest X-Ray
	Address (Zip code)	Time until first ECG
	ED Team	Number of medications given

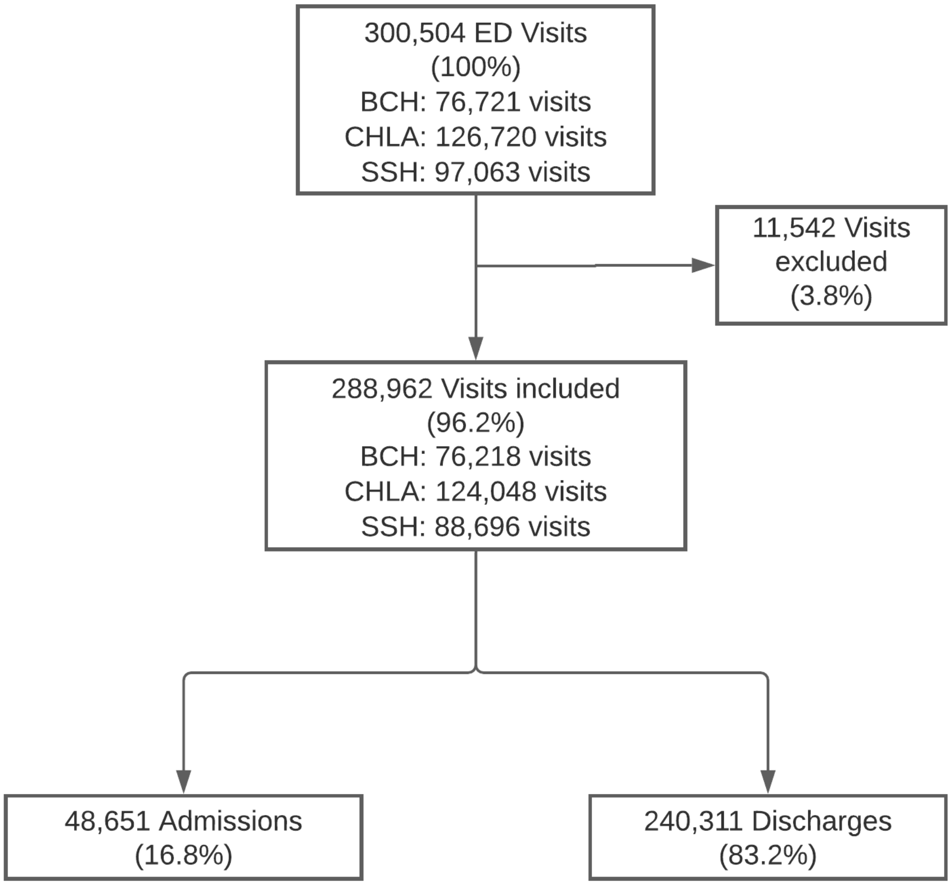

Supplementary Figure 1 - Number of visits included and excluded in the study.

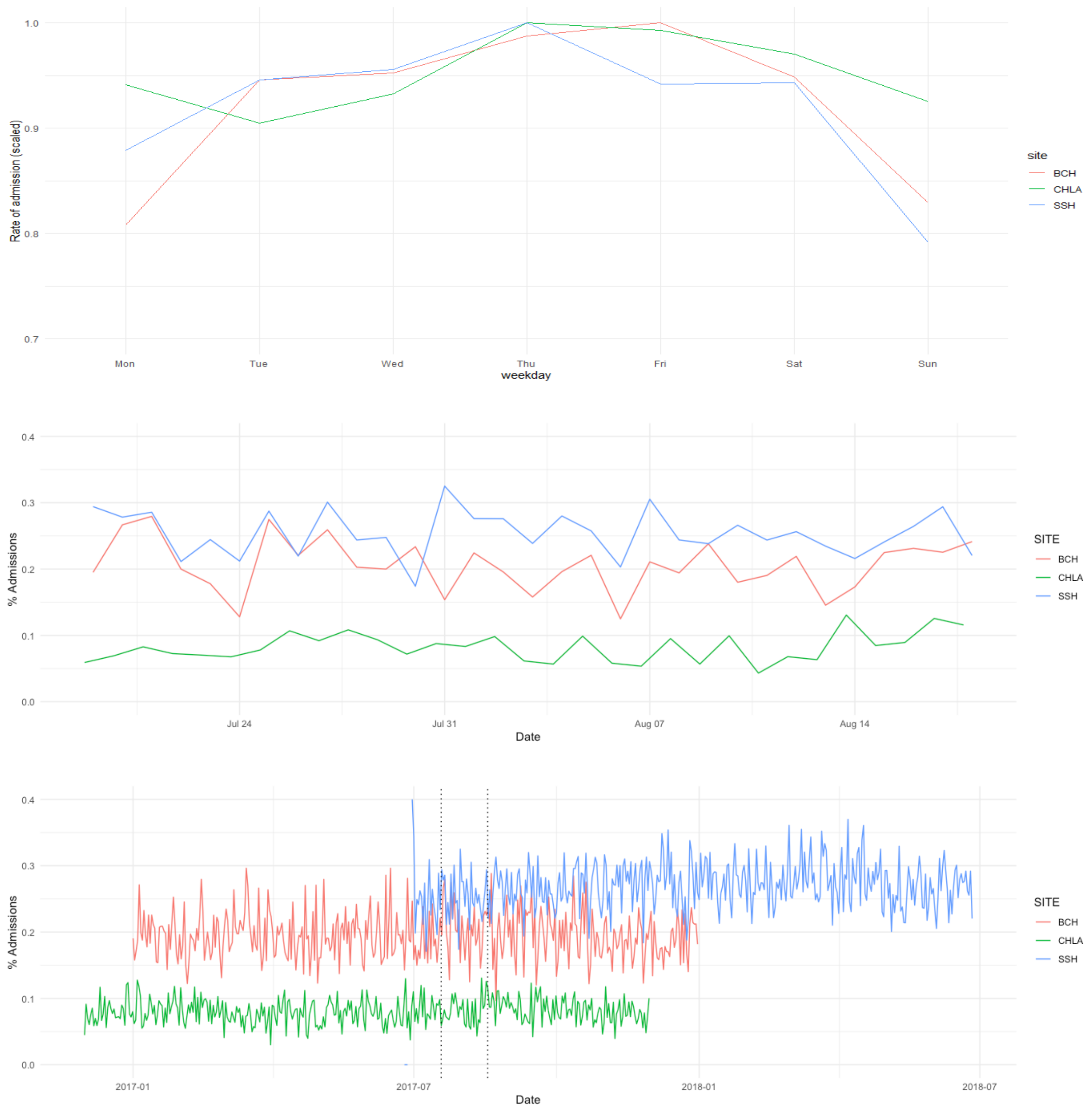

Supplementary Figure 2 - Rate of admission over time. Top: rate of admission by weekday, scaled as percent from the maximal daily admission rate. During the weekends the admission rate was lower at all sites by 1-2% ($p<0.05$). The middle plot is an enlargement of one month of the study (delimited by dotted lines in the bottom plot). Overall, the standard deviation of the admission rate across the sites ranged from 1.9% to 5.6%

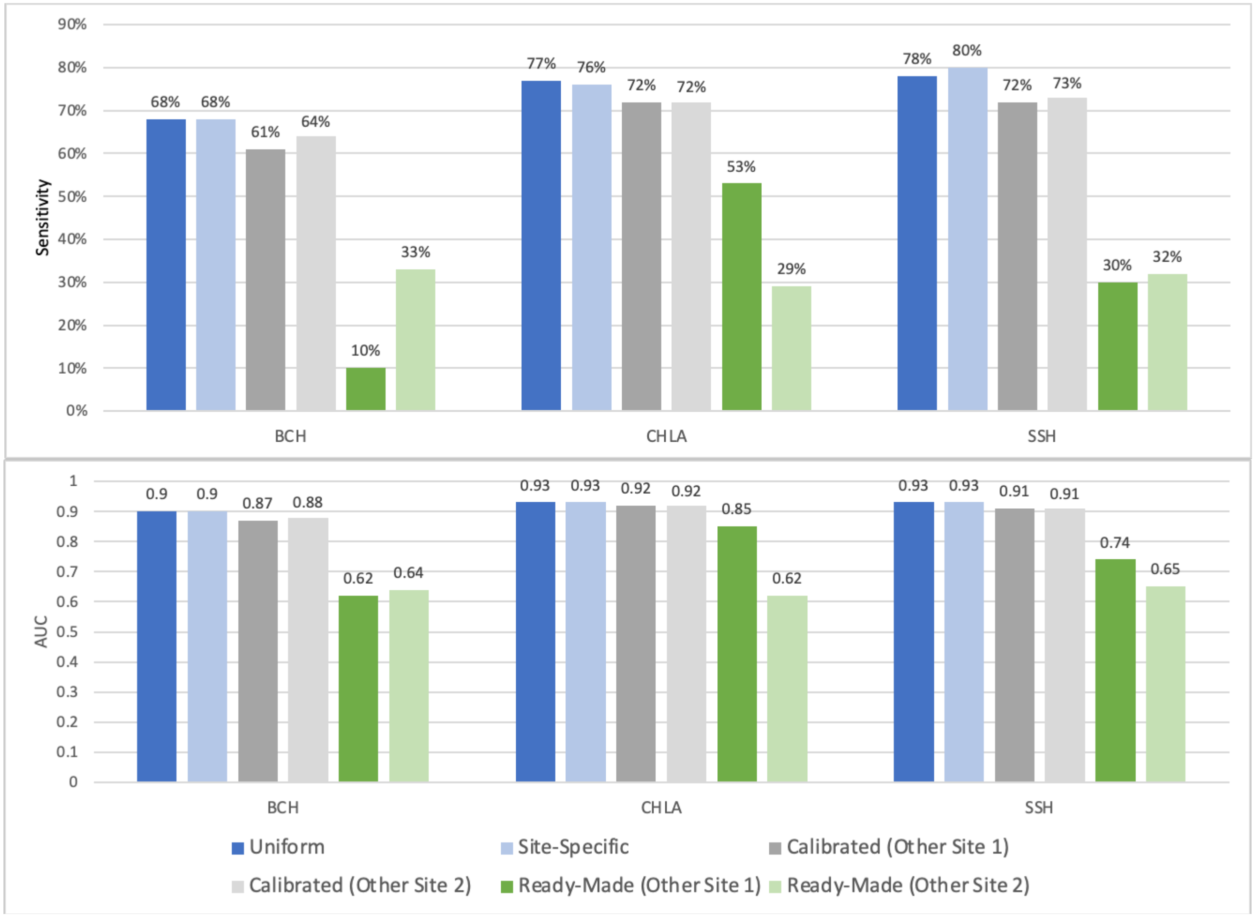

Supplementary Figure 3 - Comparison of model performance for the different modelling approaches. Top: the sensitivity of each model with a 90% specificity cutoff. Bottom: the overall AUC for each model-type and for each site.
